# Phosphorylation of the WH2 domain in yeast Las17/WASP regulates G-actin binding and protein function during endocytosis

**DOI:** 10.1038/s41598-021-88826-z

**Published:** 2021-05-06

**Authors:** J. J. Tyler, I. I. Smaczynska-de Rooij, L. Abugharsa, J. S. Palmer, L. P. Hancock, E. G. Allwood, K. R. Ayscough

**Affiliations:** Department of Biomedical Science, Firth Court, University of Sheffield, Sheffield, S10 2TN UK

**Keywords:** Biochemistry, Cell biology

## Abstract

Actin nucleation is the key rate limiting step in the process of actin polymerization, and tight regulation of this process is critical to ensure actin filaments form only at specific times and at defined regions of the cell. WH2 domains are short sequence motifs found in many different actin binding proteins including WASP family proteins which regulate the actin nucleating complex Arp2/3. In this study we reveal a phosphorylation site, Serine 554, within the WH2 domain of the yeast WASP homologue Las17. Both phosphorylation and a phospho-mimetic mutation reduce actin monomer binding affinity while an alanine mutation, generated to mimic the non-phosphorylated state, increases actin binding affinity. The effect of these mutations on the Las17-dependent process of endocytosis in vivo was analysed and leads us to propose that switching of Las17 phosphorylation states may allow progression through distinct phases of endocytosis from site assembly through to the final scission stage. While the study is focused on Las17, the sole WASP family protein in yeast, our results have broad implications for our understanding of how a key residue in this conserved motif can underpin the many different actin regulatory roles with which WH2 domains have been associated.

## Introduction

The WH2 motif is an abundant and versatile G-actin binding sequence found in a wide array of actin binding proteins^1,2^. WH2 motifs are characterised by an N-terminal amphipathic helix and a C terminal LKKV motif^1^. The N-terminal helix binds to actin at the hydrophobic cleft at the barbed end of a monomer whilst the LKKV motif extends along the monomer towards the pointed end. The interaction with the barbed end blocks addition of the WH2 bound monomer to the pointed end of a filament, potentially enhancing directional polymerisation^1^.

WH2 motifs are intrinsically disordered regions (IDRs), which adopt multiple conformations in solution and only adopt a defined structure upon binding to actin^3^. Like other IDRs the lower folding constraints allow for large variation in sequence. This, as well as the dynamic nature of the interaction between the WH2 motif and the actin monomer, helps to explain the broad array of divergent actin related processes in which WH2 motifs are implicated. These include: nucleation, filament severing, monomer sequestration, monomer delivery and regulation of barbed-end dynamics^2,4–8^. Despite the range of associated functions, multiple crystal structures show different WH2 motifs binding to actin in an almost identical manner, making the source of the functional variation unclear. However, some reports reveal that small changes in sequence can result in significant changes in activity. For example, mutation of residues within the amphipathic helix of the third WH2 motif of Spire have been shown to markedly reduce nucleation, without having a large effect on the strength of G-actin binding^9^. Similarly, the polar nature of the C-terminus of the helix in the first WH2 motif of the nucleator Cobl has been shown to be important for its nucleation activity^8^.

In terms of a role in actin filament nucleation, in some proteins, for example those of the WASP family, WH2 domains are located adjacent to sites that also bind Arp2/3. In other proteins WH2 domains are found in pairs or tandem arrays that have the potential to bind multiple actin monomers, and this binding has been proposed to support actin nucleation independently of Arp2/3^6,10–13^. The ability of the WH2 domain to bind G-actin is critical for Arp2/3-mediated actin nucleation and subsequent polymerization. However, the balance between binding of G-actin, and the subsequent ability to release this monomer to be incorporated into a growing filament is likely to be a key aspect of this mechanism, and one that has received relatively little attention^14^. It might be expected that regulating the affinity of WH2 binding for actin has the potential to play a critical role in rate of nucleation and onset of filament growth. The nucleation promotion activity of WASP family proteins is regulated by a number of mechanisms in vivo. Phosphorylation within the C-terminal intrinsically disordered region e.g. on WASP/N-WASP S483,S484/S484,S485 has been detected^15^, though to date phosphorylation has mostly been identified and considered in terms of its potential to promote nucleation by disruption of the auto-inhibitory interface between the C-terminal CA region and the GTPase binding CRIB domain^3,16,17^.

Las17 is the only WASP family protein in the budding yeast *Saccharomyces cerevisiae*. It is the primary activator of Arp2/3-driven actin nucleation, and is required for membrane invagination during endocytosis^18–20^. Las17 has a similar domain structure to mammalian WASP having an N-terminal WH1 domain, a proline rich region and a C-terminal WH2 G-actin binding motif adjacent to a central and acidic (CA) region that binds Arp2/3. Las17 does not however contain a GTPase binding region so is not considered to be regulated by binding of rho-family GTPase proteins. The yeast protein Vrp1 (homologue of mammalian WASP interacting protein, WIP) also contains WH2 domains and is proposed to act with Las17 during later stages of endocytosis^21^. The prevailing model of Arp2/3-driven actin nucleation and polymerization in cells requires binding of G-actin by the WH2 domain and delivery of this monomer to the actin pre-nucleus formed by Arp2 and Arp3 within the Arp2/3 complex. Central to the model, is that the WH2 domains both bind actin monomer and release it^22,23^. Understanding the balance between these bound and release states could help explain differences in functionality of WH2 domain containing proteins and identify key points of actin nucleation regulation.

In this study we have used mass spectrometry to analyse yeast Las17/WASP and have mapped phosphorylation sites including one at residue Ser554 within the WH2 domain. Functional analysis reveals changes in G-actin binding affinity attributable to this phosphorylation event. In cells a phospho-mimetic mutation (S554D) causes defects during the invagination and scission stages of endocytosis while, the non-phosphorylatable S554A mutation causes a strong defect at an earlier endocytic stage. Together the data indicate the importance of this WH2 motif residue for switching actin binding affinity of Las17 during distinct stages of endocytosis and might shed light on the functional variation across the spectrum of proteins carrying WH2 domains.

## Results

### Las17 can be phosphorylated in the WH2 domain

**Figure 1:**
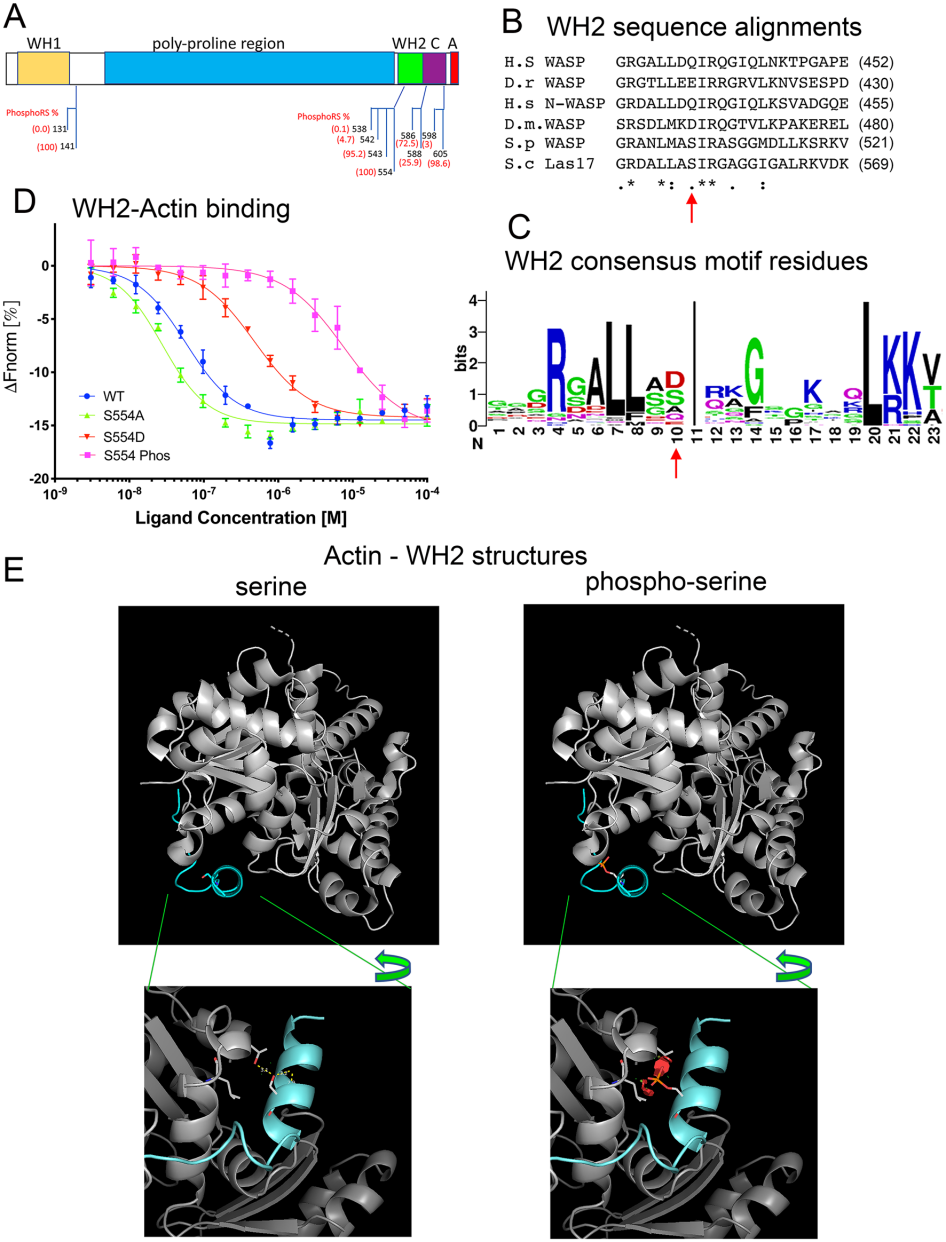
Phosphorylation of Las17 and impact on WH2 actin binding. (**A**) Mass spectrometry analysis revealed phosphorylated Las17 peptides. Most likely sites of modification indicated by phosphoRS analysis are indicated on Las17 schematic. (**B**) Alignment of WH2 domains from WASP family proteins from humans (h.s NP_000368); Danio rerio (NP_956232.1) Human N-WASP (BAA20128.1), *Drosophila melanogaster* WASP (NP_651637.1) *Schizosaccharomyces pombe* Wsp1 (AAB92587) and *Saccharomyces cerevisiae* Las17/WASP (CAA99390). Arrow indicates Serine 554—phosphorylated in *S. cerevisiae* Las17. (**C**) WH2 consensus generated using WebLOGO indicating relative frequency of specific amino acids at certain sites in a consensus generated from 43 WH2 seed sequences listed on the Pfam database. Arrow indicates position equivalent to S554 in Las17 (**D**) Thermophoresis trace to measure G-actin binding affinity of wild type, phosphorylated and mutant WH2 peptides. Peptides corresponding to amino acids 541 to 569 were used in MST to show binding to 50 nM labelled actin. The assays were carried out in G-buffer plus 0.05% tween-20. (**E**) The pdb structure (3MN5) of the 4th WH2 domain of Spire was used to model Las17 WH2 domain bound to yeast actin with and without a serine phosphorylation modelled at Ser554 (upper panels). Lower panels show an expanded view of the interfacial region between actin subdomain 3 and the Spire WH2 domain peptide. Orientation is changed (denoted by arrow) in order to show the steric clashes that are imposed by phosphorylation at S554 (red). Pair-wise overlap of atomic Van der Waals radii as predicted by PyMOL. The discs range in colour from green (slight overlap) to red (major overlap). Actin residues that are indicated with steric clashes T351 and L349 (equivalent to T353 and L351 in rabbit muscle actin).

Yeast Las17, tagged with 3xHA epitope, was immunoprecipitated from extracts, isolated from gels and following tryptic digest, analysed by liquid chromatography tandem mass spectrometry (LC–MS/MS). Two approaches were employed during the analysis, multidimensional protein identification technology (MudPIT) and neutral loss multi stage activation (MSA). Phosphorylation site localization probabilities for all peptide match spectra were calculated using the phosphoRS algorithm. Figure 1A depicts phosphorylation sites (and their probabilities) identified using this analysis. Two of these sites S586 and S588 have been previously reported^24^. A number of these sites lie in the C-terminal region of the protein with T543 just upstream of the WH2, while S554 lies within the predicted N-terminal helix forming region of the WH2 domain. The serine 554 residue is immediately upstream of the highly conserved isoleucine (I555 in Las17) which has previously been demonstrated to be key to actin binding (Fig. 1B). Analysis of the WH2 consensus sequence based on 43 seed sequences in the Pfam database (Fig. 1C) reveals only a limited range of residues at the position immediately upstream (− 1) of the conserved isoleucine. These residues are alanine, serine, glutamine, aspartate and glutamate. Phosphorylation at serine 554 in Las17 gives the possibility to switch from a small uncharged amino acid to an acidic, charged residue, thus potentially mimicking the difference between some of the residues most often found at this position. Other phosphorylation sites indicated by mass spectrometry were S586, S588 and S605. These lie within the less well conserved region between the WH2 and acidic region which, in other WASP proteins, has been associated with regulating autoinhibition^3,16,17^. Given the lack of apparent auto-inhibition in yeast Las17, and the known importance of WH2 G-actin binding, this study focused on understanding the role of S554 phosphorylation.

### Phosphorylation of S554 reduces WH2 affinity for actin

In order to understand the impact of WH2 phosphorylation at Ser554 on G-actin binding, peptides were synthesized (Las17 amino acids 541–569) and incubated with labelled G-actin. Binding affinity was analysed using microscale thermophoresis (MST). As shown in Fig. 1D, phosphorylation of the WH2 peptide significantly reduced its affinity for G-actin with calculated affinity reducing from 34 to 8000 nM.

To understand why phosphorylation is having such a profound effect, we modelled serine phosphorylation in the Las17 WH2 domain using the existing PDB structure of the fourth WH2 peptide of the actin-binding protein Spire in complex with actin. As shown in Fig. 1E, the addition of a phosphate group on the serine is likely to introduce steric hindrance between the WH2 helix and actin subdomain 3 and would therefore be unlikely to form an effective binding site.

In order to undertake studies in cells, mutations of the serine were generated, switching to the commonly used alternative residues of alanine to generate a non-phosphorylatable mutant, and aspartate to generate a phospho-mimetic residue. To facilitate subsequent interpretation of in vivo data we first undertook initial binding analyses of S554A and S554D mutant peptides using MST (Fig. 1D). The mutation S554D which introduced an acidic residue, reduced G-actin binding affinity (wt 34 nM; S554D 472 nM). This reduction in affinity of about tenfold, was not as great as observed in the phosphorylated peptide but given the smaller size and charge of aspartate relative to the phosphorylated serine this would be expected. Modelling of the WH2-actin interaction for the mutations is shown in Supplementary figure S1 and demonstrates some steric clash for the S554D but not the S554A mutation. Intriguingly, the S554A mutation also had a marked effect on binding affinity causing an increase in affinity to 7.0 nM. Thus, the S554A mutation, while it does prevent phosphorylation is not a simple equivalent to a non-phosphorylated residue. However, given the observed frequency of alanine at the − 1 position relative to the conserved isoleucine in WH2 domains (Fig. 1C), we reasoned that this mutant would allow us to investigate the impact of WH2 higher affinity actin binding further.

### S554 mutants affect actin polymerization in vitro

**Figure 2:**
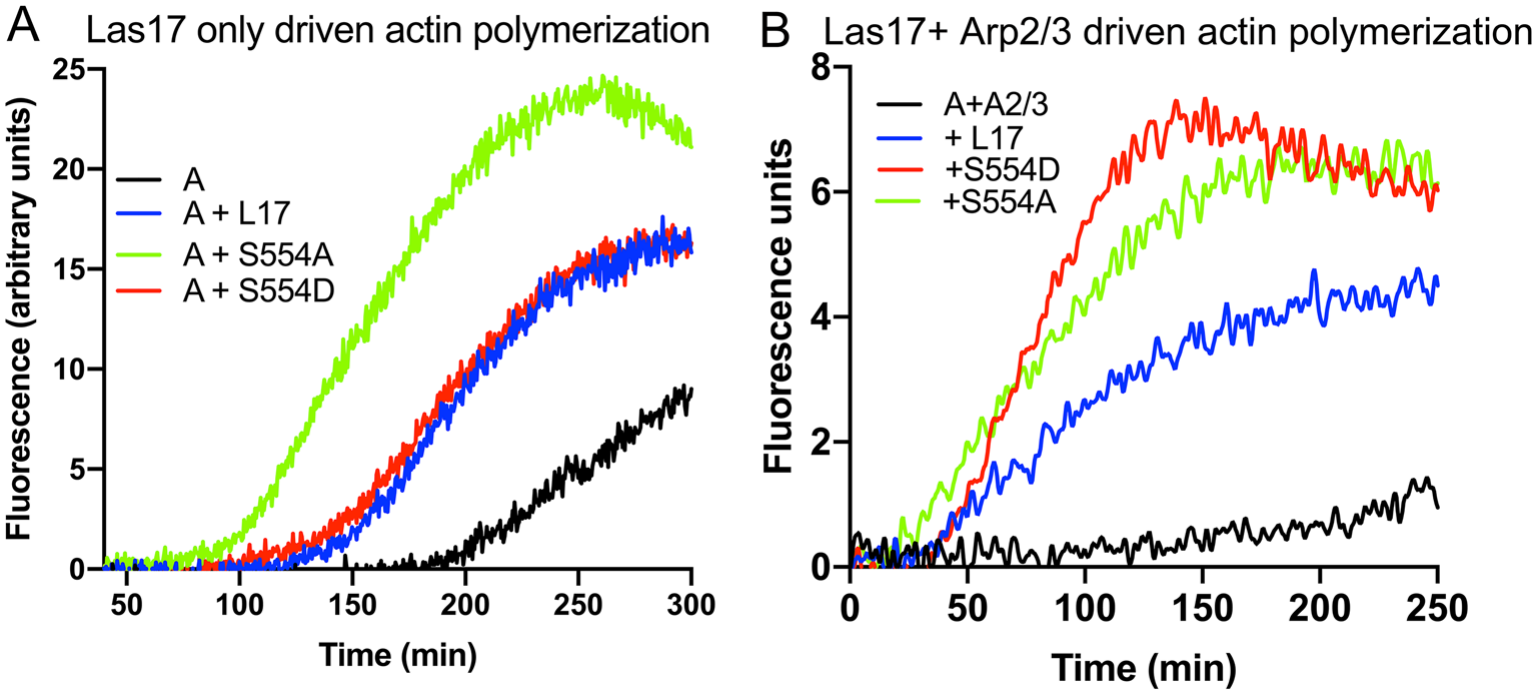
The effect of Las17 S554 mutations on actin polymerization in vitro. 0.5 µM 5% pyrene labelled actin was polymerized in the presence of G-buffer plus 0.5 × KME as described in the absence (**A**) or presence (**B**) of 5 nM Arp2/3. The effect of a Las17 fragment (amino acids 300–633) either wild-type or with an S554A or S554D mutation was investigated. Experiments were repeated at least three times and representative traces are shown. Analysis of significant differences was carried out using Uncorrected Dunn’s test and showed that in the absence of Arp2/3 the lag phase for nucleation by S554A is significantly reduced to about 40% of wt and S554D Las17 lag phase times (*p* = 0.0447, and 0.0180 respectively). In the presence of Arp2/3 the lag phase for nucleation with S554A is reduced compared to that of wt (*p* = 0.0153) There is no significant difference between the lengths of the lag phases for S554A and S554D.

Given the marked changes in affinity for the WH2 peptides and G-actin, we next investigated the impact of these amino acid substitutions on actin polymerisation in vitro using incorporation of pyrene-labelled actin as a marker of filament formation. Given our hypothesized function of Las17-mediated actin nucleation in generating filaments de novo, assays were performed with relatively low levels of actin and Las17. Under these conditions, the S554A mutant showed a significant increase in the rate of nucleation (a decrease in lag phase) compared to the S554D or wild type Las17 (Fig. 2A).

Following Las17-mediated generation of actin filaments de novo, we have proposed that Arp2/3 can then bind to these mother filaments and drive further actin nucleation and filament growth. We therefore also analysed the effect of the S554A and S554D mutants in the presence of Arp2/3. As shown in Fig. 2b, the mutants had a different effect. While the S554A still reduced the lag phase, the S554D mutant showed an enhanced elongation rate possibly because its lower affinity permits more rapid release of actin and its incorporation into filaments.

### The effect of Las17 S554 mutants on actin function in vivo

In order to investigate the physiological role of phosphorylation within the WH2 domain of Las17 in *S. cerevisiae* the S554A and S554D mutations were generated at the *LAS17* locus in the genome. Expression of both mutants rescued the temperature sensitivity of the *las17* deletion indicating at least partial functionality (Supplementary Fig. S2). Rhodamine-phalloidin staining to visualize F-actin revealed that both Las17 mutants rescued the large aggregates of actin observed in the *las17* deletion strain though the S554A mutation appeared to cause a reduction in the polarised spatial distribution of actin patches in cells (Supplementary Fig. S2).

**Figure 3:**
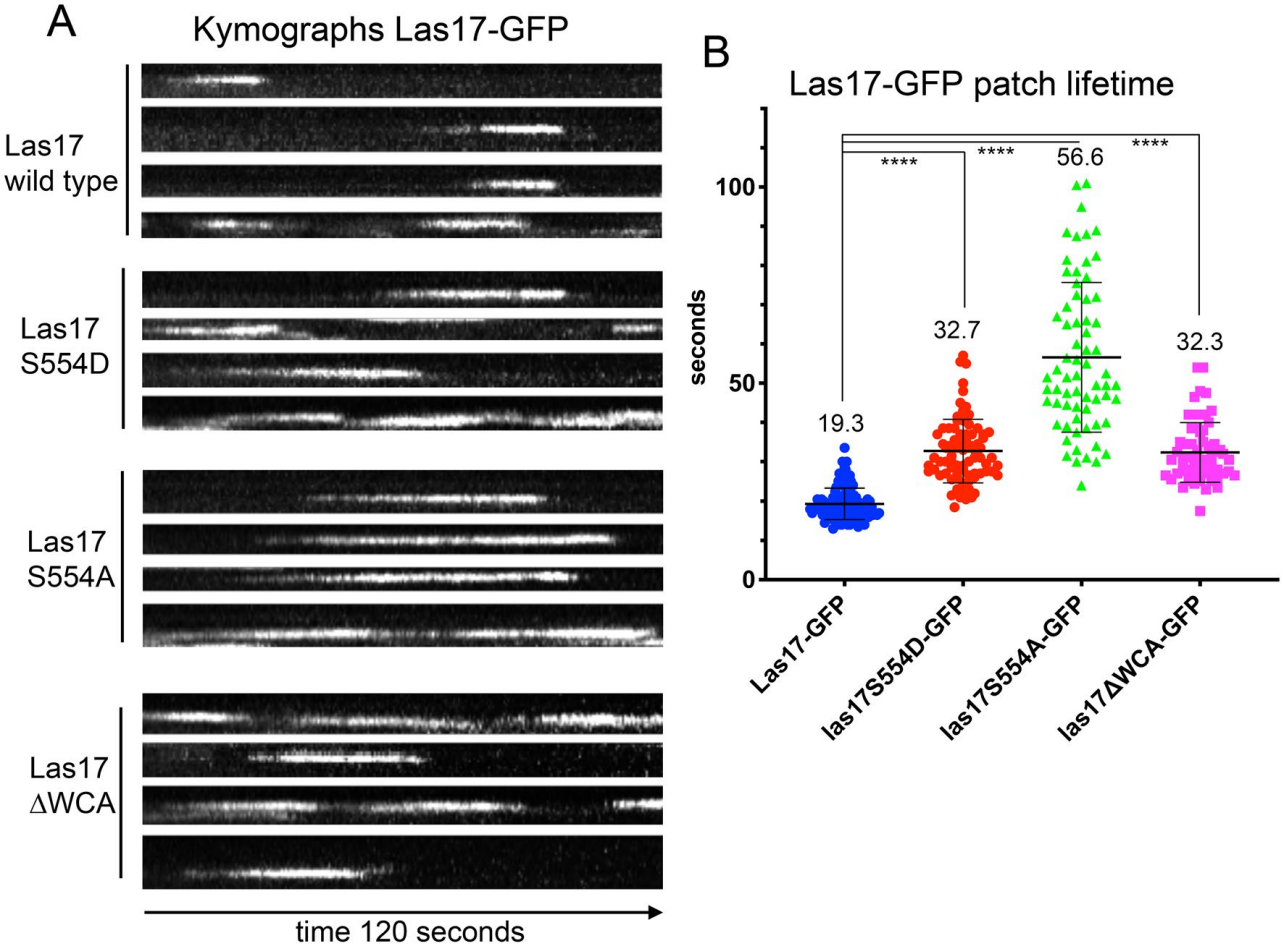
The effect of Las17S554 mutations on Las17-GFP in vivo. Wild type or mutant Las17 was tagged in the genome with a 7 × alanine linker and GFP and visualized as described. (**A**) Representative kymographs showing Las17-GFP. Vertical axis = distance from membrane, horizontal axis = time. (**B**) Lifetimes of patches were measured (combined data from from ≥ 3 experiments ≥ 5 cells on each occasion; n = 112 wt, n = 78 S554D, n = 67 S554A, n = 62 las17∆WCA). One way ANOVA with Dunnetts multiple comparison test indicates that all mutants show a significantly longer lifetime than the wild type protein n (*p* < 0.0001 in all cases). The Las17S554D and Las17∆WCA mutants.

GFP-tagged versions of the Las17 mutants were made in the genome. As shown in Fig. 3A, B both mutations caused an increase in the lifetime of Las17 at the plasma membrane compared to the wild-type tagged protein indicating that both phosphorylated and dephosphorylated states may have roles at stages of the endocytic process. Given actin affinity changes, we reasoned if the S554D mutation reduced binding markedly in vivo then it could potentially resemble mutants with a complete loss of the WCA region. To test this, a GFP tagged Las17∆WCA mutant was generated and its lifetime analysed. As shown, this truncation mutant has a very similar lifetime to the Las17 S554D mutation. Coupled with analysis of the behavior of both Las17∆WCA and of the G-actin non-binding I555D mutant^25^, this supports the idea that phosphorylation potentially acts to also reduce G-actin binding in vivo.

### The effect of the S554 mutations on individual endocytic events

The increased lifetime of Las17 mutants at the plasma membrane suggested there would be defects during endocytic progression. We therefore investigated the effect of the S554A and S554D mutations on the assembly of two reporters at endocytic sites. These were Sla1 a component of the late endocytic coat which, in wild-type cells, localizes at the same time as Las17, and Arc15 a subunit of the Arp2/3 complex which is usually recruited to sites about 10 s after Las17. The in vitro data demonstrated that the S554A mutant reduces the lag phase, indicative of a greater F-actin nucleation rate in the absence of Arp2/3 which might be expected to impact on all stages of actin function in endocytosis while the phosphomimetic mutant S554D might be predicted to have an lesser impact due to other proteins also functioning in recruitment of G-actin at the sites.

**Figure 4:**
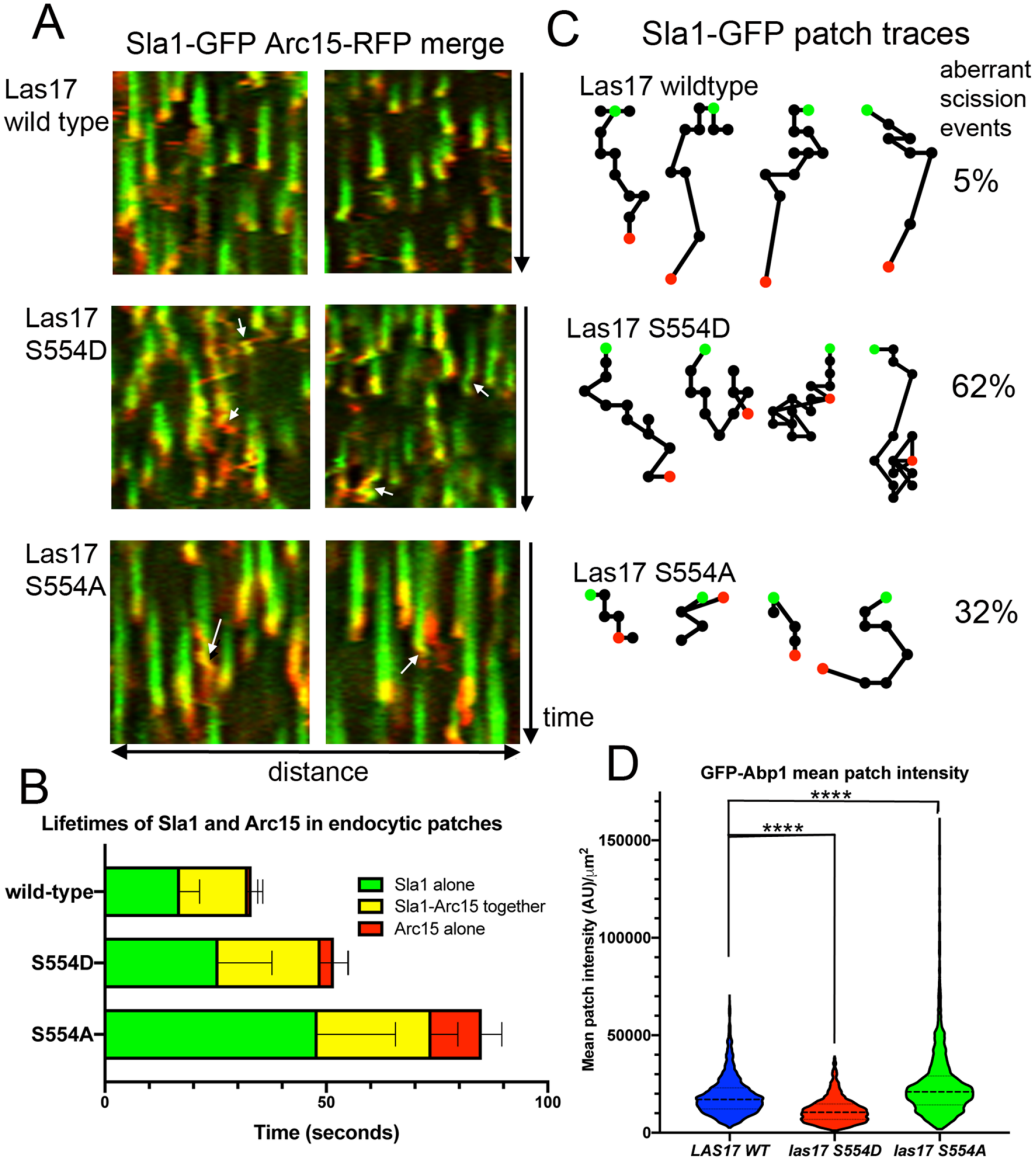
The effect of Las17S554 mutations on individual endocytic events. (**A**) Representative kymographs from the bud circumference of cells expressing wild-type or Las17S554A/D mutants tagged with Sla1-GFP (green) and Arc15 (red). As individual kymographs are from endocytic patches whole surface of the cell invaginations are shown by movement in either direction on the x axis, and retractions are characterised by a subsequent change in direction on the x axis. Arrows indicate retraction events. (**B**) Lifetimes of Sla1 and Arc15 labelled patches in wild type and mutant cells. Green indicates time of only Sla1-GFP in the patch; yellow when both Sla1 and Arc15 are present and red the time when only Arc15 is observed. Error bars show standard deviation for each lifetime. Sla1-GFP and Arc15-ChFP patch lifetimes are significantly different for both mutants compared with the wild-type judged by one way ANOVA with Dunnetts multiple comparison test (*p* < 0.0001) (**C**) Patch tracking was also used to analyse the behavior of individual endocytic patches labelled with Sla1-GFP. Green dots represent the start of tracks and are on the membrane, red dots represent the ends of tracks. Movement in the plane of the membrane is represented on the x axis, y axis represents movement perpendicular to the plane of the membrane. The proportion of events measured from movies where retraction is observed are noted to the right of the images. n = 76 wt; n = 58 S554D; n = 60 S554A. (**D**) GFP-Abp1 was used to determine whether there was an overall impact of the mutations on the level of F-actin at endocytic sites over their lifetime. Dotted line shows mean; violin plots shows total distribution of intensity data. One way ANOVA with Dunnetts test indicates the S554D mutant causes a significantly lower recruitment of GFP-Abp1 while the S554A mutant causes a significant increase in GFP-Abp1 at individual endocytic sites (*p* < 0.0001 denoted ****).

As shown in Fig. 4A,B, both mutations cause an increase in Sla1 lifetime prior to Arc15 recruitment. The effect on overall lifetime of the S554A mutation is markedly more pronounced than S554D with a long delay before Arc15 recruitment and also a much longer lifetime of Arc15. Analysis of labelled patches reveals behavioural differences in the different mutants (Fig. 4A,C). As expected the wild type patches show relatively uniform behavior with Sla1 recruitment preceding Arc15 arrival, followed almost immediately by an invagination movement. In the presence of the Las17 S554D mutant while there was an increase in Sla1 lifetime before Arc15 recruitment, the most striking change was during invagination when there was an increase in the number of failed scission events. Rather than invagination being followed by an abrupt disassembly of factors from sites (as in wild type cells) the invagination appears to persist or retract back towards the membrane (Fig. 4A, arrowheads and 4C). In the presence of the higher affinity binding S554A mutant the clearest impact was on the lifetime of Sla1 prior to Arc15 recruitment which was nearly three times longer than that measured in wild type cells. Invaginations were often shallow but retraction and other failed scission events were also observed (arrows).

To address whether the S554 mutations led to an altered level of F-actin at endocytic sites, we analysed the recruitment and intensity of Abp1-GFP signal at sites. Abp1 is an actin binding protein that requires F-actin in order to localize to sites^26^. As shown, in the accompanying movies and figure (Supplementary information and Supplementary Fig. 3), the lifetime of Abp1-GFP at endocytic sites reflects the same pattern as for Arc15, with elevated life times of localization for the mutants, especially for the S554A mutant. Both the mean and maximum intensity of patches were analysed to assess whether the level of F-actin at sites was increased. As shown (Fig. 4D), expression of the Las17 S554A mutant led to an increase in the overall mean level of F-actin at endocytic sites, though there was also a marked level of variation in patch intensity. Conversely, the phosphomimetic mutant that reduces affinity for G-actin has a lower level of F-actin at sites, supporting the idea that G-actin binding by WH2 contributes to overall recruitment of actin to endocytic sites.

Together the data demonstrates a role for phospho-regulation of Las17/WASP underpinning key stages of endocytosis. Many kinases have been associated with endocytic function in yeast but because endocytic events occur asynchronously in cells and, because kinases often function in sequence, co-dependently or redundantly it is difficult to determine impacts of specific single modification events using population based approaches such as mass spectrometry or gel analysis of Las17 from cells. In order to remove some of these complexities we generated a reporter peptide of Las17 amino acids 500–581 carrying a mutation at T543A, the only other phosphorylation site clearly identified in this region (Fig. 1A). This reporter, as well as one additionally mutated at S554A (T*S*) was expressed in cells. While the T*S reporter runs at a higher molecular weight than the T*S* double mutant indicating that phosphorylation of the fragment can occur in cells, analysis of the reporter in single kinase deletions of (*yak1, yck1, yck2, ark1, prk1, and pho85*) and double kinase deletions (*ark1∆,prk1∆; yck1∆yck2ts*) in our hands revealed no inhibition of S554 phosphorylation.

## Discussion

While phosphorylation of WASP family members has been reported to influence function, this is the first report of a regulatory phosphorylation event within the actin-binding WH2 domain^15–17,27^. The dramatic shift in actin binding affinity on phosphorylation of the serine within the WH2 N-terminal helix region of Las17 is substantial and may in part explain the breadth of functions associated with WH2 domains in proteins. Three major findings are reported in this study: 1 an acidic residue at this site in WH2 reduces affinity for actin, reduces actin levels at endocytic sites and impacts on success of scission events. 2. an alanine residue at this site has higher actin binding affinity but delays early stages of endocytic progression. 3. phosphorylation of serine adjacent to the conserved isoleucine in the alpha helix of the WH2 domain in Las17 significantly reduces actin binding. Together the data suggest that switching of Las17 phosphorylation states may allow progression through distinct phases of endocytosis from site assembly through to the final scission stage.

In yeast, Las17 arrives at plasma membrane sites about 15 s prior to the well-studied nucleating complex Arp2/3 and before the only other WH2 domain containing protein Vrp1^28–30^. We have previously proposed that Las17 alone is capable and responsible for generating mother filaments de novo at endocytic sites^25,31^.

The S554 mutants had clear phenotypic differences when expressed in cells. For S554D a reduced ability to recruit G-actin is reflected in the lower intensity of F-actin at endocytic sites. This lower level of F-actin did not however appear to be strongly inhibitory during the initial or invagination stages of endocytosis though intriguingly, the scission stage of endocytosis did appear to be significantly defective with the majority of invaginations showing failed scission. Fitting with previous data^25^ this suggests that regions of Las17 other than WH2 are key to recruitment of actin at early stages of endocytosis. This Arp2/3-independent activity leads to generation of F-actin sufficient to then recruit Arp2/3 and other actin binding proteins such as Vrp1and the type I myosins. In turn this facilitates membrane invagination, with the reduced actin binding affinity of S554D/phosphorylation potentially beneficial to facilitate the rapid growth of Arp2/3 generated actin filaments. The scission defect is particularly interesting and suggests that WH2 dephosphorylation is required to meet an additional requirement for actin recruitment, possibly for further filament nucleation or to facilitate tension generation, to complete vesicle scission. A possible role for actin during vesicle scission has been suggested from electron microscopy studies which have detected Las17 localization mid-way down invaginations prior to scission and from an analysis of yeast dynamin function and in studies linking dynamin/Vps1 vesicle scission with actin function^29,32^.

The phenotype of the S554A mutation is more severe and highlights potential impacts of non-phosphorylatable mutations sometimes causing distinct phenotypes themselves. However the prevalence of alanine at this specific position in the N-terminal helix of a significant number of WH2 domains meant that there was value in determining the effect of such a mutation within a physiological system. Despite the S554A mutation enhancing actin binding affinity and actin nucleation in vitro, the in vivo impact was to markedly inhibit the endocytic process both by delayed recruitment of Arp2/3 and of the inhibition of the subsequent invagination stage. It is possible that the increased actin affinity means that Las17 remains associated with the nascent filament for longer and thereby precludes effective recruitment of Arp2/3. Alternatively, there could be increased F-actin polymerization at endocytic sites but this actin might be structured or organized in such a way that appropriate levels of Arp2/3 to drive invagination are not recruited.

Thus within the context of yeast, we hypothesize that phosphorylation at Ser554 may provide a regulatory switch between a phosphorylated weak binding state that favours Arp2/3-dependent nucleation during invagination to a stronger (though still polar, serine residue) binding that more effectively supports Arp2/3-independent nucleation that functions at initial and final stages of the process.

In terms of broader implications of this study, we have analysed other WH2 domains and note that there is a restricted number of residues that occupy the position -1 to the highly conserved isoleucine in the N-terminal WH2 helix (Fig. 1C). Our in vitro binding data and that from yeast would indicate that the nature of this residue has very marked implications for the function of the protein in which it resides. Those proteins with alanine at the site may bind G-actin more strongly and, if there are tandem similar domains this might be critical to allow a stable nucleus to be formed and for new filaments to be formed. In contrast proteins that harbor an acidic residue at this site are likely to have weaker binding. Such activity might be less effective for forming a nucleus but might be more optimal to support rapid filament growth as the monomer are likely to be released more readily into filaments. Supporting this idea, Cobl which is viewed as a strong nucleator, has three tandem actin-binding WH2 domains^13^. All of these have alanine at the site adjacent to the isoleucine. Spire on the other hand which has a weaker nucleating activity has Glu, Asp, Phe and Ser at its predicted WH2 domains^9,11^. The only other protein in *S. cerevisiae* with WH2 domains is verprolin (Vrp1), which is proposed to function only in the invagination, stages of endocytosis concomitant with Arp2/3 activity. The two Vrp1 WH2 domains both contain Asp at the position adjacent to the conserved isoleucine supporting the idea that reduced affinity is associated with enhanced activity in the presence of Arp2/3.

Overall this study reveals the possibility of a phosphorylation mediated regulation of yeast WASP function to support distinct stages of endocytosis. It also more broadly highlights the importance of a key residue within WH2 domains that might allow more general prediction of the activity of a actin binding protein.

## Methods

Chemicals and media were obtained from Sigma-Aldrich, Fisher Scientific, or Formedium.

### Yeast growth and strain generation

Yeast strains used in this study are listed in supplemental Table 1. Yeast plasmids used: pKA88 carrying GFP tagged Abp1^26^, pKA606 carrying wild type Las17^25^; mutations in pKA606: pKA1087 with Las17 S554A and pKA1088 with S554D. Unless stated otherwise, cells were grown with rotary shaking at 30 °C in liquid YPD medium (1% yeast extract, 2% Bacto-peptone, 2% glucose supplemented with 40 μg/ml adenine) or in synthetic medium (0.67% yeast nitrogen base, 2% glucose) with appropriate supplements. All strains carrying fluorescent tags have growth properties similar to control strains. Point mutations in *LAS17* were generated using site directed mutagenesis (Agilent). DNA cassettes carrying mutations for integration were transformed into KAY1801. Mutant colonies were counter-selected on minimal medium, containing 0.005% uracil and 0.1% 5'-Fluoroorotic Acid (5-FOA; Melford laboratories). Allele exchange, in growing Ura3-5-FOA resistant colonies, was confirmed by PCR and sequencing.

### Fluorescence microscopy

For live-cell imaging, cells expressing tagged proteins were visualized after growing to an early log phase. Analysis was carried out at room temperature (22 °C). Imaging of GFP-tagged Sla1 and Abp1 proteins expressed singly was performed with 0.5 s exposure and 1 s time-lapse. Sla1-GFP and Arc15-mCherry co-localisation was performed with 0.5 s exposure for both fluorophores, and 2 s time-lapse. Microscopy used Olympus IX-81 inverted microscope with DeltaVision RT Restoration Microscopy System (100 × /1.40 NA oil objective), Photometrics Coolsnap HQ camera and Image capture performed using SoftWoRx image analysis and model-building application (Applied Precision Instruments, Seattle). All image data sets were deconvolved using the SoftWoRx application. Time-lapse of wild-type and mutated Las17-GFP was acquired using OMX DeltaVision V4 and a 60 × /1.42 NA objective. Images were taken simultaneously on separate scientific complementary metal oxide semiconductor (sCMOS) cameras (30 ms exposure). Seven 250 nm sections were acquired every 0.5 s (241 time points). The stacks were then deconvolved and processed, using SoftWorx. Protein localisation and lifetime was analysed from those projections. Fiji software was used to assemble movies and kymographs, to determine GFP-Abp1 patches intensity and area, and for individual spots tracking. Statistical analysis of lifetimes, patches intensity and area was performed using Graphpad Prism software.

### Protein purification and assays

Las17 fragments were expressed as GST fusion proteins as described previously^25^. Briefly plasmids were transformed into Lucigen OverExpress C41(DE3) cells and transformants selected on an agar plate containing ampicillin. Transformed colonies were grown in 2 L of 2xYT medium for 5 h or until mid-log phase. The cell cultures were then induced with 1 mM IPTG for 5 h. Pelleted cells were then frozen at − 20 °C until use. To purify the expressed protein cells were thawed in PBS containing protease inhibitors (Roche complete inhibitor cocktail) and 2 mM DTT. Cells were broken by sonication for 3 × 30 s on ice, and cell debris pelleted by centrifugation at 20,000 × *g* for 30 min. The supernatant was then supplemented with an additional 300 mM NaCl and mixed with 100 µl of Glutathione Sepharose beads which had been pre-equilibrated with buffer A (PBS plus an additional 300 mM NaCl). GST tagged protein was allowed to bind for 30 min at 4 °C with rotation. Unbound protein was then washed from the beads by a series of washes of 3 × 15 ml each of buffer B (buffer A plus 0.1% Tween), and then 3 × 15 ml PBS. Washed beads were then equilibrated in Prescission protease cleavage buffer (50 mM Tris–HCl, pH 7.0 (at 25 °C), 150 mM NaCl, 1 mM EDTA, 1 mM dithiothreitol). Las17 protein was cleaved from the GST tag by incubation with 10units of Prescission Protease for 6 h at 4 °C. Cleaved Las17 was buffer exchanged into G-buffer (2 mM Tris pH 8.0, 0.2 mM CaCl2, 0.2 mM ATP, 0.5 mM DTT) using a PD10 buffer exchange column and frozen in aliquots until required. Protein concentration was determined by comparison with a known protein standard on SDS PAGE.

Peptides used, Las17(501–528), were synthesized by Pi Proteomics (Huntsville, AL). Rabbit skeletal muscle actin was purified and gel filtered as described^31^. Briefly, acetone powder (5 g) prepared from fresh rabbit leg muscle was hydrated in G-buffer for 30 min, spun and filtered through glass wool and 0.2 µM filters, and then polymerised by the addition of KCl and MgCl_2_ for 30 min at room temperature followed by 30 min a 4 °C with gentle stirring. Polymerised actin was pelleted by centrifugation at 34,900 × *g* for 2 h before being resuspended in G-buffer and dialysed against G-buffer for 48 h to depolymerise with buffer changes twice daily. Finally monomeric actin was separated from dimers and short oligomers by gel filtration in a 1 m x 26 mm sephadex column in G-buffer. Peak and post-peak actin fractions containing actin monomers were stored at 4 °C for up to two weeks. Pre-peak actin fractions were pooled, dialysed against G-buffer without DTT, and made to 20uM before being polymerised by the addition of 100 mM KCl, 2 mM MgCl_2_. Pyrene (4 mg dissolved in 400 µl DMSO) was added to the F-actin and stirred overnight to allow binding. The pyrene actin was then depolymerised by dialysis against G-buffer and unbound pyrene was removed by gel filtration in a 1000 mm × 26 mm sephadex column. Pyrene incorporation was determined by measuring absorbance at 290 nm and 344 nm.

For fluorimetry assays, 370 μl assays used 0.5 μM actin. Pyrene-actin was added to 5%, mixed thoroughly, and added to the fluorimetry cuvette. Polymerization salts, 10 × KME (500 mM KCL, 10 mM MgCl_2_, 10 mM EGTA, 100 mM Tris–HCl [pH 8.0]) were used at final concentration of 0.5 × KME before reading fluorescence. Polymerization was observed in a Cary Eclipse fluorometer (emission 364 nm, slit 10 nm round; excitation 385 nm, slit 20 nm) as described previously^31^. Unless otherwise stated Las17 fragments were used at 25 nM and Arp2/3 (obtained from Cytoskeleton Inc, USA) at 2 nM.

Microscale Thermophoresis (MST) was used to measure binding affinities with actin as previously described^31^. Briefly, rabbit muscle actin in G-buffer without DTT was labelled using a Monolith NT™ protein labelling kit RED-Malemide (NanoTemper Technologies, Munich, Germany). Las17 was prepared as two-fold serial dilutions and an equal volume of labelled actin was added to each reaction (Final buffer conditions: G-buffer + 0.05% Tween). Each reaction was then loaded into Monolith NT.115 Premium Treated Capillaries (NanoTemper Technologies) and thermophoresis was measured in a Monolith NT.115 Microscale Thermophoresis device (NanoTemper Technologies). Thermophoresis was measured at 20% red LED power and 40% IR laser power at 22 °C. The Fnorm was calculated from the ratio of fluorescence before heating and after the new equilibrium had been reached. Fnorm was then plotted against Las17 concentration to give the Las17 dependent change in thermophoresis. This was described by the ‘Kd fit’ model on the provided software to give Kds for actin binding to Las17 fragments.

### Analysis of Phosphorylation of S554 in vivo

A Las17 gene fragment encompassing sequence for amino acids 500–581 was cloned into a vector pKA526 (pTPI1-mcs-3xHA) at BamH1 and Sal1 sites to allow generation of Las17(500–581) with a 3 × HA tag. To facilitate analysis of phosphorylation of only the S554 phosphorylation event, the T543 residue was mutagenized to alanine, using a QuikChange mutagenesis kit and appropriate oligonucleotides, generating pKA1264. Mutagenesis was verified by sequencing. A further reporter with S554 also mutagenized to alanine was also created (pKA1266). The plasmids were transformed into wild type or strains harbouring deletions of various kinases that have been associated with endocytosis. Strains carrying plasmids were selected and grown to log phase. 5 ml cells were broken in lysis buffer in the presence of protease and phosphatase inhibitors (50 mM Tris–HCl pH 7.5, 100 mM NaCl, 0.1% Triton X-100, 0.1% β-mercaptoethanol, 1 mM EGTA, 2.5 mM EDTA, 1 mM PMSF, protease inhibitor cocktail without EDTA [Roche], 10 μM Leupeptin, 1 μM Pepstatin-A, PhosSTOP cocktail [Roche], 1 mM NaF, 0.5 mM Na_3_VO_4_ ) using small scale bead-beating. EZ view red anti-HA agorose beads (Merck) were added to the supernatant and incubated overnight at 4 °C with rotation. Beads were washed with lysis buffer, then resuspended in 2 × gel sample buffer and boiled. Proteins were separated using SDS-PAGE (15% polyacrylamide) in the presence of 200 µM PhosTag (Alpha labs) and blotted onto membrane. Membranes were probed with anti-HA antibodies (rat; Sigma Aldrich).

### Structural modelling

Las17 residues 546–567 (WH2 domain) were structurally modelled using the 4^th^ WH2 domain of Spire obtained from an actin-bound structure (pdb 3MN5). Structural modelling was carried out using the homology-modelling server found on the SWISS-MODEL website (https://swissmodel.expasy.org/) which gave an acceptable QMEAN^33–37^. Amino acid substitution to phosphoserine was achieved using the SwissSidechain PyMOL plugin (v2)^38^. The mutagenesis wizard tool in PyMOL (version 2.4.1) was used to suggest how residue substitutions within the WH2 domain may result in spatial clashing with actin residues^39^.

## Supplementary information

Supplementary Information 1.

Supplementary Information 2.

Supplementary Video 1.

Supplementary Information 3.

## Data Availability

The majority of data generated or analysed during this study are included in this published article (and its Supplementary Information files).
