# Supplementary material for: Phosphorylation of the WH2 domain in yeast Las17/WASP regulates G-actin binding and protein function during endocytosis
Source: Sci Rep. 2021 May 6;11:9718. doi: 10.1038/s41598-021-88826-z (PMC8102491; doi:10.1038/s41598-021-88826-z)

Supplemental Information

Phosphorylation of the WH2 domain in yeast Las17/WASP regulates G-actin binding and protein function during endocytosis

Tyler, J.J.[1‡], Smaczynska de Rooij, I.I.[1‡], Abugharsa, L.[1‡], Palmer, J.S.[1], Hancock, L.P. [1], Allwood, E.G. [1], and Ayscough, K.R. [1*]

Supplementary Methods. Supplementary Table S1. **Yeast strains used in this study**

Strain	Genotype	Note
KAY389	*MAT***a** *his3-Δ200 leu2-*3,112 *ura3-*52 *trp1-1 lys2-801*	[40]
KAY1801	*MAT***a** *his3-Δ200 leu2-*3,112 *ura3-*52 *trp1-1 lys2-801 las17Δ::URA3*	This study
KAY1859	*MAT***a** *his3-Δ200 leu2-*3,112 *ura3-*52 *trp1-1 lys2-801 las17 S554D*	This study
KAY1860	*MAT***a** *his3-Δ200 leu2-*3,112 *ura3-*52 *trp1-1 lys2-801 las17 S554A*	This study
KAY400	*MAT***a** *his3-Δ200 leu2-*3,112 *ura3-*52 *trp1-1 lys2-801 SLA1-GFP::TRP1*	[40]
KAY1865	*MAT***a** *his3-Δ200 leu2-*3,112 *ura3-*52 *trp1-1 lys2-801 las17Δ::URA3 SLA1-GFP::TRP1*	This study
KAY1876	*MAT***a** *his3-Δ200 leu2-*3,112 *ura3-*52 *trp1-1 lys2-801 las17 S554D SLA1-GFP::TRP1*	This study
KAY1877	*MAT***a** *his3-Δ200 leu2-*3,112 *ura3-*52 *trp1-1 lys2-801 las17 S554A SLA1-GFP::TRP1*	This study
KAY1878	*MAT***a** *his3-Δ200 leu2-*3,112 *ura3-*52 *trp1-1 lys2-801 Sla1-GFP::TRP1 ARC15-mCherry::HIS3*	[31]
KAY1881	*MAT***a** *his3-Δ200 leu2-*3,112 *ura3-*52 *trp1-1 lys2-801 las17 S554D SLA1-GFP::TRP1 ARC15-mCherry::HIS3*	This study
KAY1882	*MAT***a** *his3-Δ200 leu2-*3,112 *ura3-*52 *trp1-1 lys2-801 las17 S554A SLA1-GFP::TRP1 ARC15-mCherry::HIS3*	This study
KAY1912	*MAT***a** *his3-Δ200 leu2-*3,112 *ura3-*52 *trp1-1 lys2-801 LAS17-7xAla-GFP::kanMX6*	This study
KAY1913	*MAT***a** *his3-Δ200 leu2-*3,112 *ura3-*52 *trp1-1 lys2-801 las17 S554A LAS17-7xAla-GFP::kanMX6*	This study
KAY1914	*MAT***a** *his3-Δ200 leu2-*3,112 *ura3-*52 *trp1-1 lys2-801 las17 S554D LAS17-7xAla-GFP::kanMX6*	This study
KAY1591	*MAT***a** *his3-Δ200 leu2-*3,112 *ura3-*52 *trp1-1 lys2-801 las17 ΔWCA-7xAla-GFP::kanMX6*	This study
KAY446	MAT*a, his3Δ1, leu2Δ0, met15Δ0, ura3Δ0*	Invitrogen
KAY1542	MAT*a, his3Δ1, leu2Δ0, met150Δ, ura3Δ0, yck1Δ::KanMX*	Invitrogen
KAY1543	MAT*a, his3Δ1, leu2Δ0, met150Δ, ura3Δ0, yck2Δ::KanMX*	Invitrogen
KAY1544	MAT*a, his3Δ1, leu2Δ0, met15Δ0, ura3Δ0, yak1Δ::KanMX*	Invitrogen
KAY1685	MAT*a, his3Δ1, leu2Δ0, met15Δ0, ura3Δ0, pho85Δ::KanMX*	Invitrogen
KAY1671	MAT*a, his3, leu2, ura3-52, yck1Δ::URA3, yck2-2^{ts}*	[41]
KAY376	*MATa, his3-Δ200, leu2-3,112, ura3-52, trp1-1, lys2-801, ark1Δ::HIS3*	[42]
KAY381	*MATa, his3-Δ200, leu2-3,112, ura3-52, trp1-1, lys2-801, prk1Δ::LEU2*	[42]
KAY379	*MATa, his3-Δ200, leu2-3,112, ura3-52, lys2-801, prk1Δ::LEU2, ark1Δ::HIS3*	[42]

Supplementary Video. GFP-Abp1 in cells expressing wild type and mutant Las17.

Supplementary Data file

Mass spec data files used for identification of S554 phosphorylation

Supplementary Figures

Supplementary figure S1. Modelling of the WH2-actin interaction interface in the S554A and S554D Las17 mutants. The pdb structure (3MN5) of the 4th WH2 domain of Spire was used to model Las17 WH2 domain bound to actin with alanine or aspartate modelled at Ser554 instead of serine (upper panels). Lower panels show an expanded view of the interfacial region between actin subdomain 3 and the Las17 WH2 domain peptide. Orientation is changed in order to show the steric clashes that are imposed by phosphorylation at S554 (red). Pair-wise overlap of atomic Van der Waals radii as predicted by PyMOL. The discs range in colour from green (slight overlap) to red (major overlap).

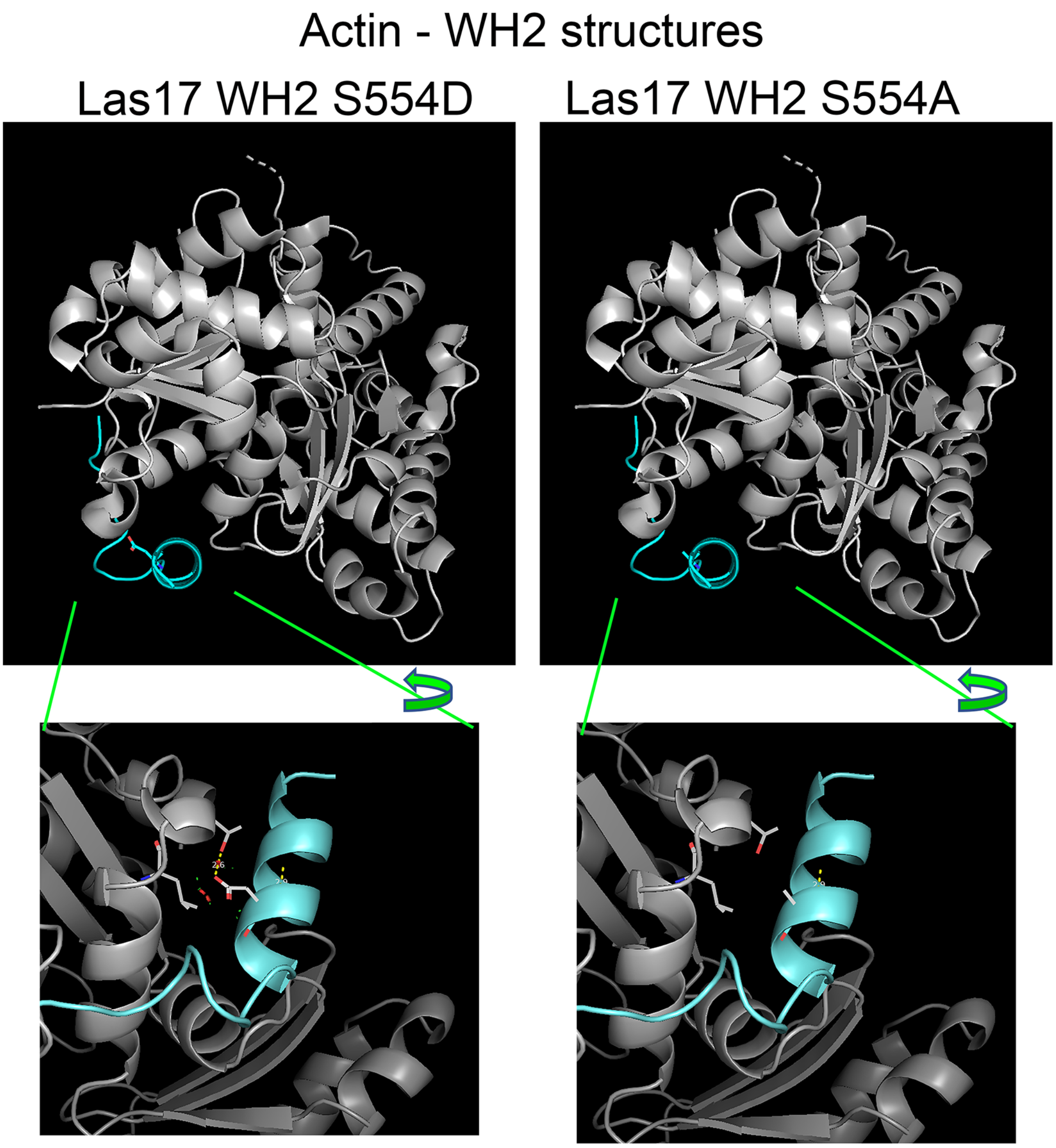

Supplementary Figure S2. The effect of Las17 S554 mutants on growth and actin organization. (A) The impact of growth on plates for cells expressing wild type, mutant or deleted for Las17 was assessed. As shown, both Las17 S554A and Las17 S554D expressing cells grow at 37°C as does the wild type. (B) Rhodamine phalloidin binding was used to assess F-actin localization and organization in cells as described [34] (C) Quantitation of organization reveals, wild type cells mostly show polarized patches of actin as well as actin cables (3 rpts; n>100 cells for each strain). Cells lacking *las17* do not show cables or normal patches, instead they have large aggregates of actin. The S554D mutant had a relatively normal distribution and appearance of actin while the S554A mutant has mostly depolarized (but not aggregated) actin. Scale bar 2 μm.

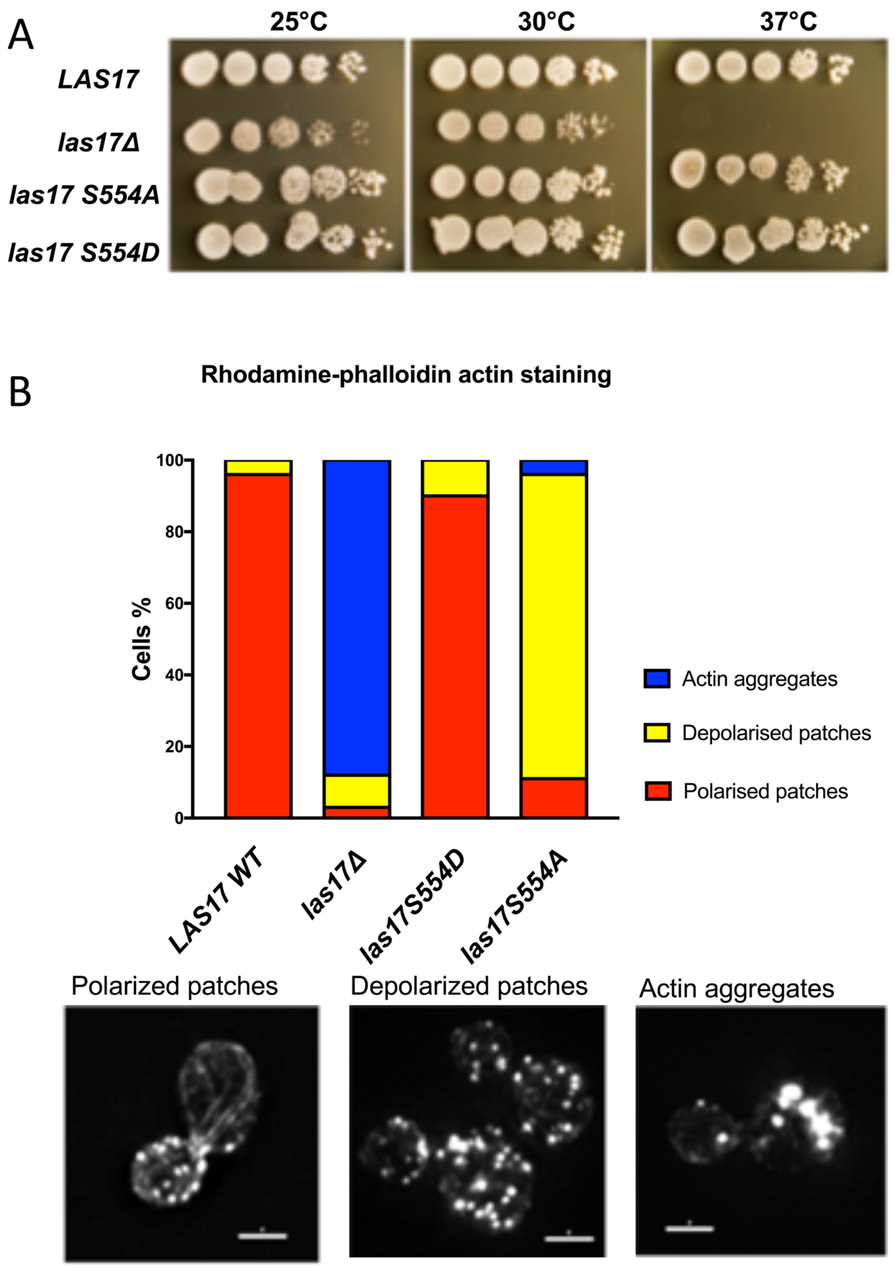

Supplementary Figure S3. The effect of Las17 S554 mutants on GFP-Abp1 at endocytic sites (A) Kymographs showing movement of GFP-Abp1 patches over time. (B) Lifetime of GFP-Abp1 patches in wild type and Las17 S554 mutant cells. **** indicates p value <0.0001; n=106 wt; n=84 S554D; n=89 S554A.

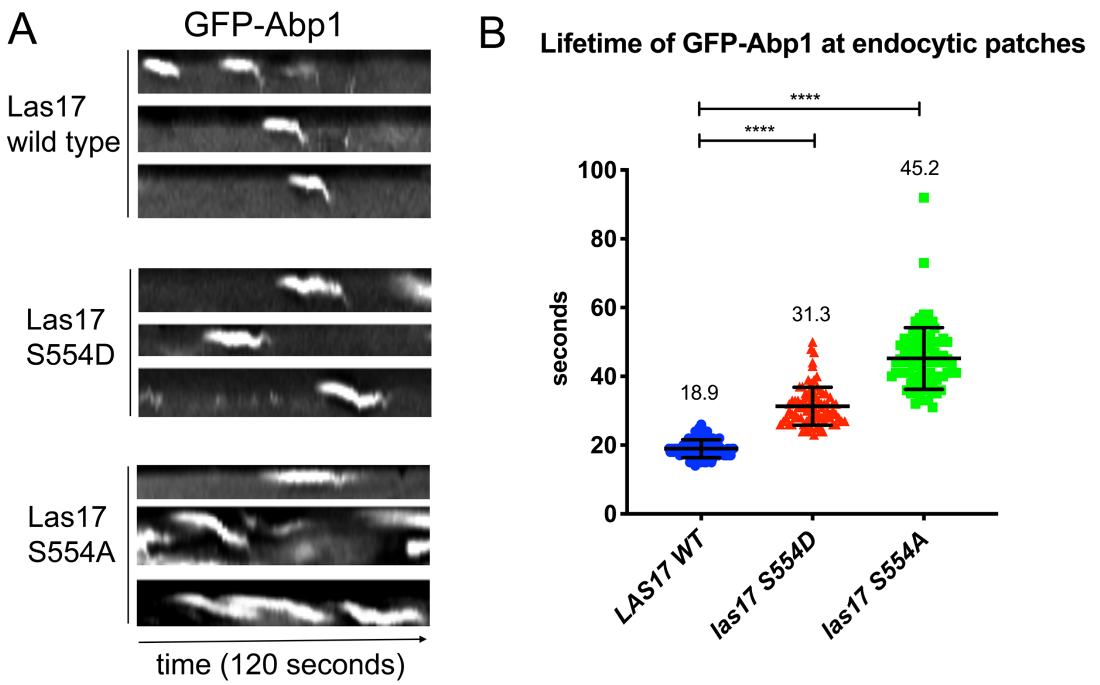

Supplementary Figure S4. The effect of kinase mutations on a Las17 reporter. A Las17 gene fragment was cloned to allow generation of Las17(500-581) with a 3x HA tag. To facilitate analysis of phosphorylation at only S554, the T543 residue was mutagenized to alanine (reporter denoted T*). Another reporter with S554 also mutagenized to alanine was also created (T*S*). The plasmid reporters were transformed into wild type or strains harbouring deletions of various kinases that have been associated with endocytosis. Strains carried full deletions with the exception of *yck1 yck2ts* as *yck1*Δ and *yck2*Δ deletions are synthetically lethal when combined [41]. This strain was grown at 37°C to abrogate yck2 function before making extracts for pull downs. EZ view red anti-HA agarose beads (Merck) were added to supernatant from lysed log phase cells, incubated overnight then beads were washed with lysis buffer and resuspended in gel sample buffer. Proteins were separated using SDS-PAGE (15% polyacrylamide) in the presence of 200 μM PhosTag (Alpha labs) and blotted onto membrane. Membranes were probed with alkaline phosphatase conjugated anti-HA antibodies (rat; Sigma Aldrich). Bands marked: 3xHA-Las17(500-581) is the reporter protein; LC denotes antibody light chain from the pull down; n.s is a non-specific band in the pull downs.

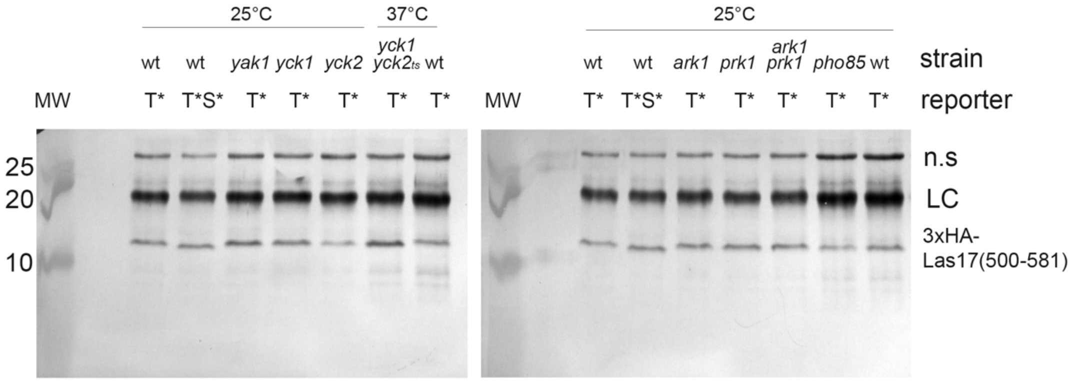

Supplement: Supplementary file 4 — Supplementary Information 3. [file 41598_2021_88826_MOESM4_ESM.pdf]
